# Identification of Core Genes Related to Progression and Prognosis of Hepatocellular Carcinoma and Small-Molecule Drug Predication

**DOI:** 10.3389/fgene.2021.608017

**Published:** 2021-02-23

**Authors:** Nan Jiang, Xinzhuo Zhang, Dalian Qin, Jing Yang, Anguo Wu, Long Wang, Yueshan Sun, Hong Li, Xin Shen, Jing Lin, Fahsai Kantawong, Jianming Wu

**Affiliations:** ^1^Department of Medical Technology, Faculty of Associated Medical Sciences, Chiang Mai University, Chiang Mai, Thailand; ^2^School of Pharmacy, Southwest Medical University, Luzhou, China; ^3^International Education School, Southwest Medical University, Luzhou, China; ^4^Education Ministry Key Laboratory of Medical Electrophysiology, Sichuan Key Medical Laboratory of New Drug Discovery and Drugability Evaluation, Luzhou Key Laboratory of Activity Screening and Drugability Evaluation for Chinese Materia Medica, Southwest Medical University, Luzhou, China

**Keywords:** hepatocellular carcinoma, prognosis, weighted gene co-expression network analysis, risk score, multivariate cox

## Abstract

**Background:**

Hepatocellular carcinoma (HCC) is one of the most leading causes of cancer death with a poor prognosis. However, the underlying molecular mechanisms are largely unclear, and effective treatment for it is limited. Using an integrated bioinformatics method, the present study aimed to identify the key candidate prognostic genes that are involved in HCC development and identify small-molecule drugs with treatment potential.

**Methods and Results:**

In this study, by using three expression profile datasets from Gene Expression Omnibus database, 1,704 differentially expressed genes were identified, including 671 upregulated and 1,033 downregulated genes. Then, weighted co-expression network analysis revealed nine modules are related with pathological stage; turquoise module was the most associated module. Gene Ontology (GO) and Kyoto Encyclopedia of Genes and Genomes pathway analyses (KEGG) indicated that these genes were enriched in cell division, cell cycle, and metabolic related pathways. Furthermore, by analyzing the turquoise module, 22 genes were identified as hub genes. Based on HCC data from gene expression profiling interactive analysis (GEPIA) database, nine genes associated with progression and prognosis of HCC were screened, including *ANLN*, *BIRC5*, *BUB1B*, *CDC20*, *CDCA5*, *CDK1*, *NCAPG*, *NEK2*, and *TOP2A*. According to the Human Protein Atlas and the Oncomine database, these genes were highly upregulated in HCC tumor samples. Moreover, multivariate Cox regression analysis showed that the risk score based on the gene expression signature of these nine genes was an independent prognostic factor for overall survival and disease-free survival in HCC patients. In addition, the candidate small-molecule drugs for HCC were identified by the CMap database.

**Conclusion:**

In conclusion, the nine key gene signatures related to HCC progression and prognosis were identified and validated. The cell cycle pathway was the core pathway enriched with these key genes. Moreover, several candidate molecule drugs were identified, providing insights into novel therapeutic approaches for HCC.

## Introduction

Liver cancer has been the sixth most commonly diagnosed cancer and ranks as the third leading death cause of cancer, with approximately 841,000 new incidences and 782,000 deaths yearly around the world (Bray et al., 2018), of which hepatocellular carcinoma (HCC) accounts for up to 90% of all primary liver malignancies, posing a major health problem (Villanueva, 2019). Moreover, with a 5-year survival of 18%, HCC ranks the second most lethal cancer (Jemal et al., 2017). Although survival rates of HCC patients have substantially improved from new therapeutic strategies (Liu et al., 2015), many HCC patients still face high long-term mortality, recurrence, drug resistance, and serious side effects (Cheng et al., 2009; Zhu et al., 2017). Abnormal expression of mRNAs plays critical roles in cancer etiology (Ruggero, 2013). Recent studies have reported that dysregulated mRNAs can be used to screen potential biomarkers in cancer prognosis (Li et al., 2019). Therefore, more effective prognostic biomarkers for HCC progression are urgently needed to assist the development of novel therapeutic targets to reduce mortality and improve prognosis.

During the last decades, advances in gene chips and high-throughput sequencing techniques have been widely used to screen key genes associated with cancer progression and prognosis by using biological big data and bioinformatics (Kandoth et al., 2013; Vogelstein et al., 2013). With these methods, researchers discovered five genes, *PCNA*, *RFC4*, *PTTG1*, *H2AFZ*, and *RRM1*, that were associated with the progression and prognosis of HCC (Kong et al., 2019). In addition, *FAM83D*, *TGFB1*, and *ADRB2* were shown to be associated with several malignant features and overall survival of HCC inpatients (Coulouarn et al., 2008; Wu et al., 2016; Liu et al., 2019). Consequently, the promising results of *in silico* analysis prompted us to conduct more exploration. Weighted gene co-expression network analysis (WGCNA), an R package, is an effective systematic bioinformatics algorithm that clusters highly co-expressed gene modules. Candidate biomarkers of therapeutic targets can be screened based on the correlation between phenotypes and these gene modules (Langfelder and Horvath, 2008). WGCNA has been successfully applied to the identification of biomarkers in renal cancers (He et al., 2017), pancreatic cancer (Giulietti et al., 2018), and breast cancer (Bao et al., 2019). Therefore, WGCNA should be applicable to HCC to help us understand the mechanism of tumorigenesis and progression and identify highly related gene biomarkers as potential prognostic factors or as therapeutic targets. However, to date, results for HCC have been limited or inconsistent because of the high false-positive rates in single cohort analysis studies and sample heterogeneity. Consequently, few reliable biomarkers for HCC have been identified.

**FIGURE 1:**
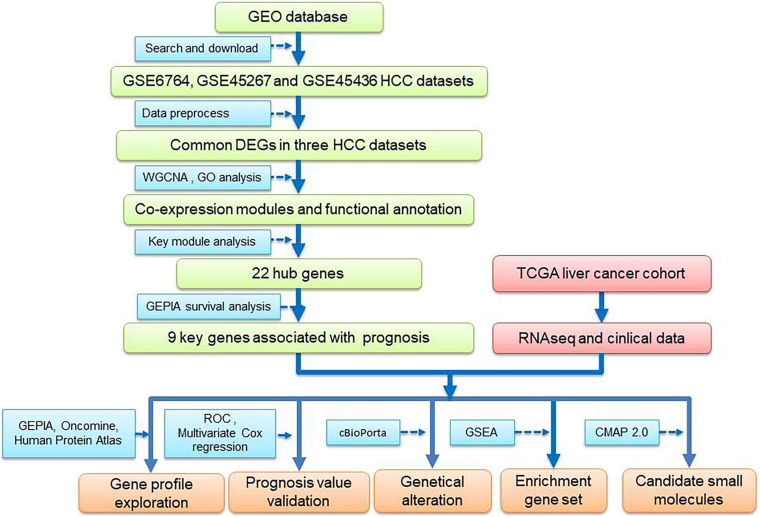
Flowchart of data analysis procedure: data collection, processing, analysis, and validation.

To overcome these limitations, multiple HCC cohort datasets and comprehensive bioinformatics analysis in a training–validation manner was used in the present study (Figure 1). Briefly, to avoid false-positive results, gene expression data from three HCC datasets from Gene Expression Omnibus (GEO) was analyzed in combination, and the common differentially expressed genes (DEGs) were identified. Furthermore, a prognostic gene module was identified by WGCNA, and along with survival analysis, a 9-gene prognostic prediction system was established. In addition, the prediction model was further validated using The Cancer Genome Atlas (TCGA) HCC dataset. Moreover, we identified several potential small-molecule drugs for HCC treatment using a connectivity map (CMap) database and analyzed the dysregulated genes in the key modules.

## Materials and Methods

### Gene Expression Dataset Collection

The HCC datasets of GSE6764, GSE45267, and GSE45436 were obtained from NCBI GEO^1^ (Supplementary Table 1). GSE6764 consists of expression data from 35 HCC and 10 normal liver samples, GSE45267 consists of data from 41 HCC and 46 normal samples, and GSE45436 consists of data from 93 HCC and 41 normal samples. DEGs were screened based on the three gene expression datasets. GSE6764 was used to construct WGCNA for this study. Level-3 RNA-sequencing data, clinical features information, and survival data of patients were obtained from Genomic Data Commons (GDC) TCGA Liver Cancer by using UCSC Xena browser^2^ for validation of hub genes.

### Data Preprocessing and Differentially Expressed Gene Screening

For preparing microarray data from GEO, probes were annotated to genes according to platform annotation profiles, and a median polish algorithm was applied for mapping multiple probes into gene symbols. The linear models for microarray data package of R were applied for DEG identification in comparisons between HCC samples and normal liver samples (Diboun et al., 2006). Cutoff criteria for screening DEGs were | fold change| > 1.5 and adjusted *P* value < 0.05.

### Construction of the Weighted Gene Co-expression Network

A weighted gene co-expression network was generated based on the protocol of WGCNA (Langfelder and Horvath, 2008). First, common DEGs were clustered to check if there were any outlier samples. Second, a soft threshold power β was identified by the function pickSoftThreshold. Third, to measure the gene biological similarity, the adjacency matrix was converted into a topological overlap matrix (TOM) for describing the degree of association between genes, and the corresponding dissimilarity (1-TOM) was used to cluster genes into gene modules through average linkage hierarchical clustering, with a minimum cluster size of 30 for avoiding abnormal modules in the dendrogram. Finally, the modules with highly correlated genes were also clustered and merged with a cutoff height of 0.25.

### Identification of Modules With Clinical Significance

The module eigengene (ME), which is the main element of a module, represented the entire characteristics of module genes. First, the correlation between MEs and clinical features of HCC stage was assessed by the Pearson test to identify the relevant gene modules. Then, gene significance (GS) and module significance (MS) were calculated. GS represents the correlation between gene expression and HCC stage. MS is the average GS for all the genes in a module (Langfelder and Horvath, 2008). Of all the modules, the module with first-ranked MS values was considered as the most significant module against HCC stage.

### Functional Enrichment Analysis

In order to explore the potential mechanism of genes in the module most related to HCC stage, we uploaded all genes in the module into database for annotation, visualization, and integrated discovery (DAVID^3^) (Dennis et al., 2003). Gene Ontology (GO) functional enrichment analysis and Kyoto Encyclopedia of Genes and Genomes (KEGG) pathway enrichment were performed. False discovery rate (FDR) < 0.05 was regarded as significant.

### Identification and Validation of Key Genes

The genes with the highest correlations in the module most related to HCC stage were defined as key genes. In this study, key genes were screened according to the criteria of cor.gene Module Membership (MM) > 0.9 and GS > 0.6. Then, expression profiles of hub genes in HCC were validated in the Gene Expression Profiling Interactive Analysis (GEPIA), Oncomine, and Human Protein Atlas databases. The top ranked genes of the hub genes having significant results with survival analysis were identified as key genes in HCC tumorigenesis. The diagnostic value of the key genes was verified by a receiver operating characteristic (ROC) curve and progression analysis using HCC TCGA data. Kaplan–Meier analysis of overall survival and disease-free survival was performed to assess the survival impact from these key genes. Moreover, a multivariate Cox regression model analysis was performed to calculate the risk score using a key gene expression signature. The risk score for each patient is defined as follows, risk score = $\sum_{i\,1}^{n}\left(c\,o\,e\,f_{i}^{*}\,\,E\,x\,p\,r_{i}\right)$, where Expr_i_ is the expression level of the genes in sample *i*, and coef_i_ is the Cox coefficient of gene i.

### Genetical Alteration Profiles of Key Genes

The cBioPortal^4^, an open online large-scale cancer genomics dataset, provides access to explore, visualize, and download multidimensional cancer genomic data (Cerami et al., 2012). In the present study, cBioPortal was used for exploring genetic alterations of key genes.

### Gene Set Enrichment Analysis of Key Genes

To further explore the potential function of the selected key genes, Gene Set Enrichment Analysis (GSEA) v4.0.3 was used to perform GSEA based on HCC TCGA data [49]. C2.cp.kegg.v7.0.symbols.gmt was chosen as a reference gene set from the MSigDB database^5^. Terms with FDR < 0.05, Gene size ≥ 10, and | enrichment score (ES) | > 0.65 were identified.

### Related Small-Molecule Compound Screening

Connectivity map^6^ (Lamb et al., 2006) was used to identify small-molecule drugs for potential HCC treatment. CMap compares gene signatures with a gene expression profile database of several cell lines after treatment with more than 1,000 compounds mostly approved by the United States Food and Drug Administration. First, we built a genes signature based on the DEGs (| log2FC| ≥ 1, FDR < 0.05) in the key module. Secondly, we upload this signature into the dataset of CMap. Connectivity scores, ranging from −1 to 1, were calculated, representing similarity of the query to each of the CMap signatures. The negative connectivity score represents that the drug can reverse input characteristics. Then, we identified compounds with negative connectivity scores, which indicate the potential therapeutic value.

## Results

### Gene Screening of Hepatocellular Carcinoma

**FIGURE 2:**
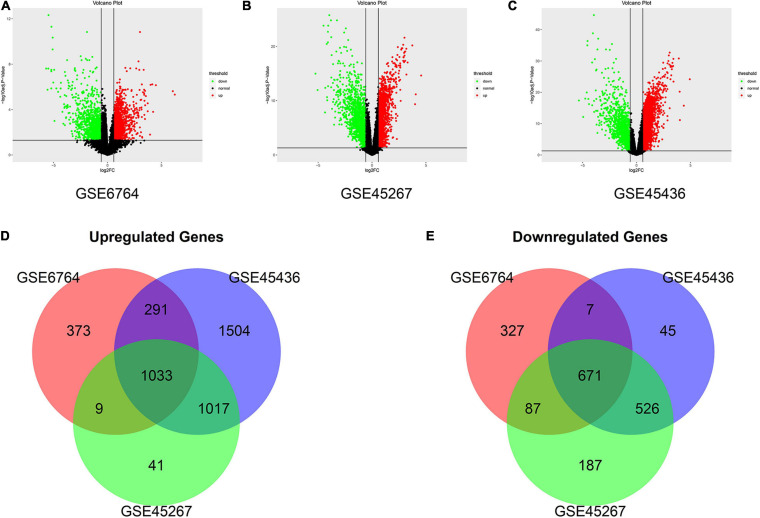
Identification of the differentially expressed genes (DEGs) between hepatocellular carcinoma (HCC) samples and normal liver samples in three datasets. **(A–C)** The volcano plots show the DEGs in GSE6764 **(A)**, GSE45267 **(B)**, and GSE45436 **(C)**, respectively. The green data points represent downregulated genes. The red data points represent upregulated genes. **(D,E)** Venn diagram demonstrates overlapped upregulated genes **(D)** and downregulated genes **(E)** in three datasets (| fold change| > 1.5 and adjusted *P* value < 0.05).

In this study, we screened DEGs between HCC samples (*n* = 169) and non-cancerous liver samples (*n* = 97) from three GEO datasets, GSE6764, GSE45267, and GSE45436. As shown in the volcano map (Figures 2A–C), all DEGs from the three datasets were identified (| fold change| > 1.5 and adjusted *P* value < 0.05). After being overlapped, we found 1,704 common DEGs, including 671 upregulated and 1,033 downregulated genes (Figures 2D,E).

### Construction of the Co-expression Network of Hepatocellular Carcinoma

**FIGURE 3:**
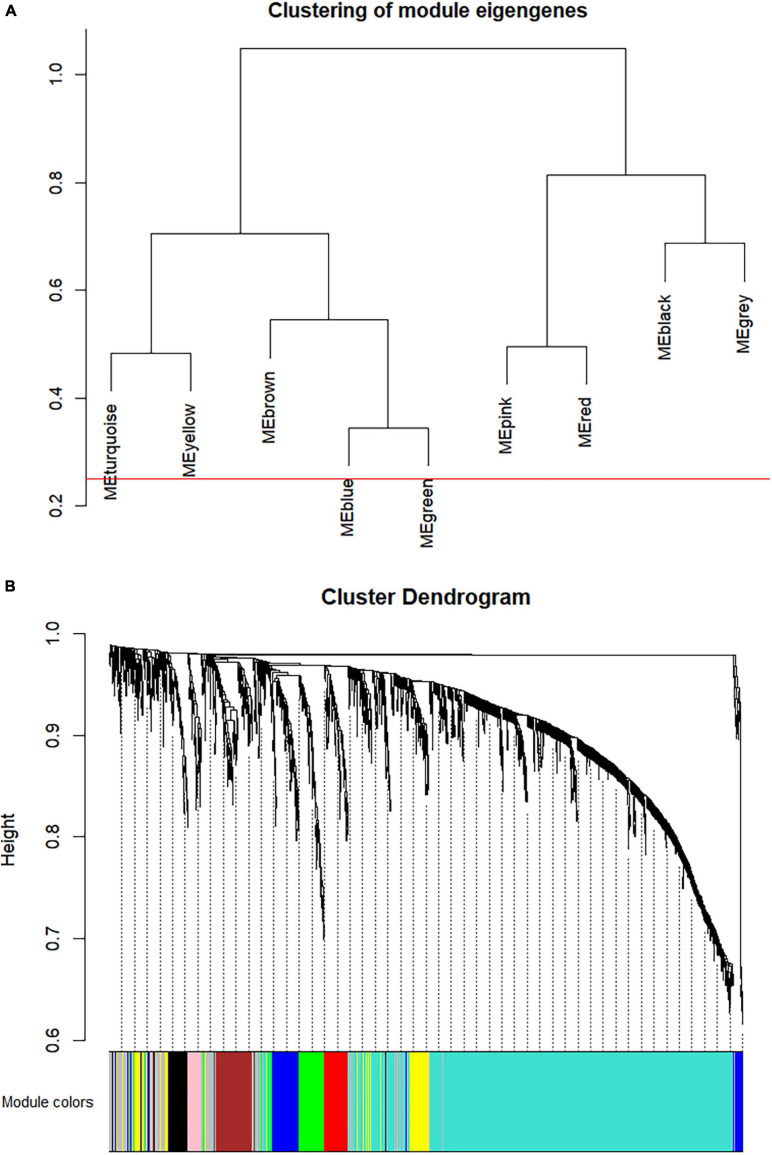
Construction of co-expression modules *via* weighted gene co-expression network analysis (WGCNA). **(A)** The hierarchy cluster dendrogram of module eigengenes. **(B)** The cluster dendrogram of the overlapped differential expression genes in GSE6764. Each piece of the leaves in the cluster dendrogram stands for a gene, and the colors below represent co-expression modules.

The co-expression analysis included 35 HCC samples with pathological stage information in the GSE6764 dataset (Supplementary Figure 1A). All 35 samples satisfied the quality assessment criteria using the WGCNA R package for co-expression analysis. To ensure a scale-free network, power of β = 4 (scale free *R*^2^ = 0.92) was selected as the soft-thresholding parameter (Supplementary Figures 1B–E). Using average linkage hierarchical clustering, nine co-expression modules were identified (Figures 3A,B). There were 62 genes in the black module, 113 genes in the blue module, 113 genes in the brown module, 91 genes in the green module, 37 genes in the pink module, 67 genes in the red module, 942 genes in the turquoise module, and 105 genes in the yellow module. The 174 genes that could not be clustered in any specific module were put into the gray module and removed in subsequent analyses.

### Identification of Key Modules

**FIGURE 4:**
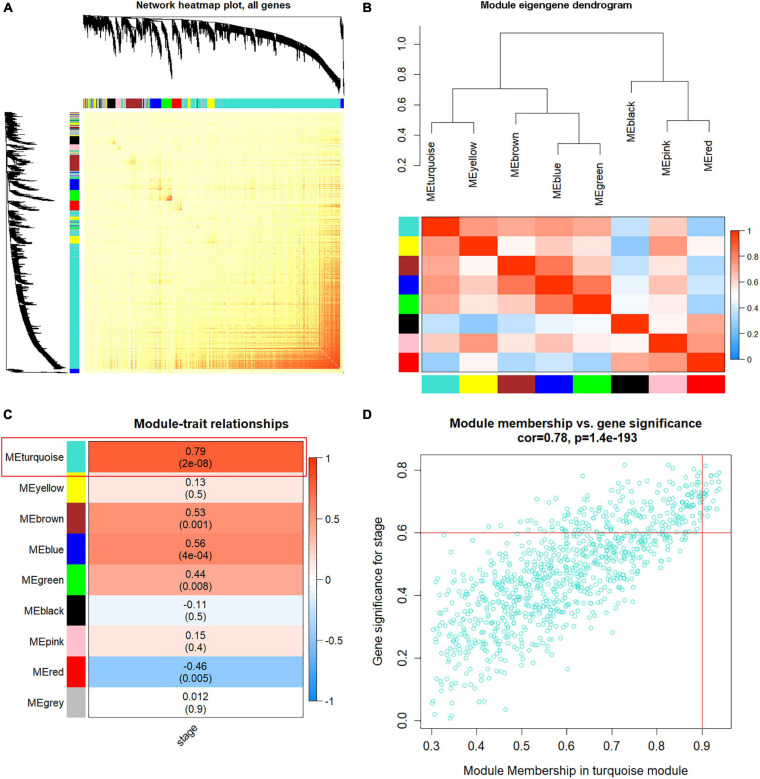
Weighted co-expression gene network construction and hub module selection. **(A)** The heatmap describes the topological overlap matrix (TOM) of all genes in weighted gene co-expression network analysis (WGCNA). Light yellow color represents a low overlap, and darker red represents higher overlap. **(B)** Eigengene dendrogram and eigengene adjacency heatmap depict the co-expression modules generated in the clustering analysis. **(C)** Heatmap of module-hepatocellular carcinoma (HCC) stage, red color for positive correlation, and blue color for negative correlation. **(D)** Scatter plot of module eigengenes based on genes in the turquoise module.

To analyze the correlation of the nine co-expression modules, the network heatmap and eigengene dendrogram were generated (Figures 4A,B), showing that eight gene modules (gray was not included) were independent of each other and mainly clustered into two groups. Moreover, with module–trait relationships, the turquoise module showed the highest correlation with HCC tumor stage compared with other modules (Figure 4C). Thus, we identified the turquoise module as the most relevant to HCC progression for subsequent analyses (Supplementary Table 2). Scatterplot of GS vs. MM (Figure 4D) shows the high correlation between MM module and GS in the turquoise. Based on the threshold that MM > 0.8 and GS > 0.6, 22 genes highly related to turquoise module were identified as hub genes (Figure 4D).

### Functional Annotation for the Turquoise Module

**FIGURE 5:**
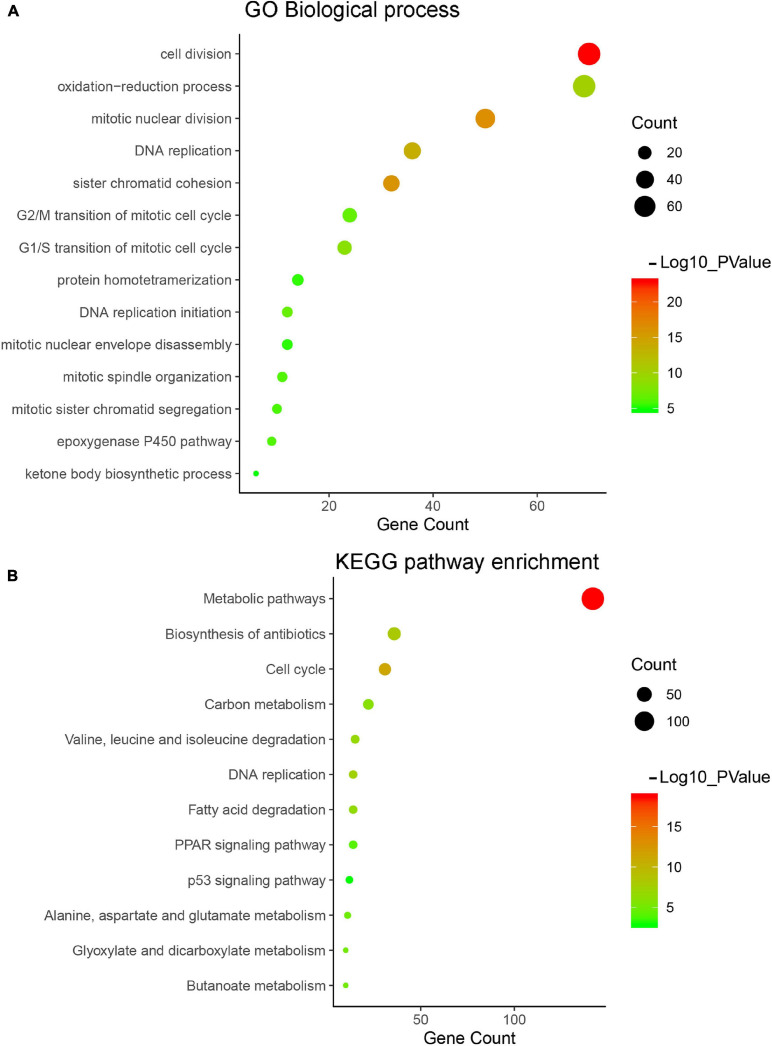
Gene Ontology (GO) and pathway enrichment analysis of turquoise module. **(A)** GO analysis of turquoise module genes. **(B)** Kyoto Encyclopedia of Genes and Genomes (KEGG) pathway analysis of turquoise module genes.

Gene Ontology and KEGG pathway enrichment were applied for genes in the turquoise module to explore potential biological significance related to HCC. Biological process of GO enrichment showed that genes in the turquoise module were mainly related to cell division, DNA replication, cell cycle, and metabolic related pathway, which played an important role in tumorigenesis of HCC (Figure 5A). The result of KEGG pathway enrichment analysis showed that the most significant pathway was metabolic pathway, the other significant pathways included cell cycle, DNA replication, etc. (Figure 5B).

### Detection and Validation of Key Genes

**FIGURE 6:**
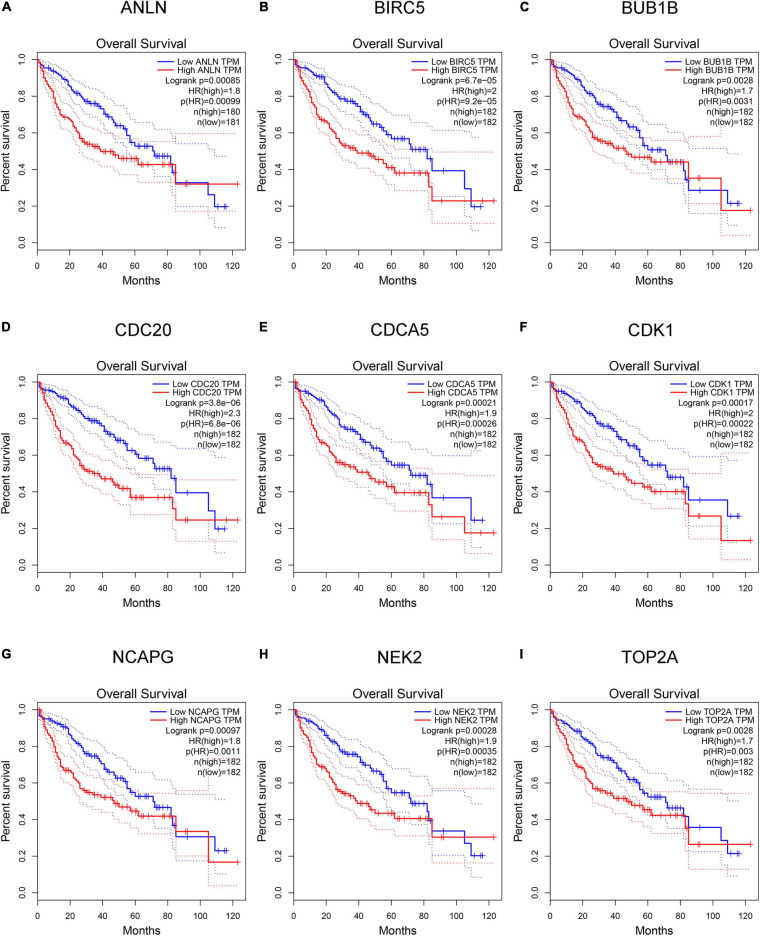
Kaplan–Meier plot of overall survival analysis of the nine key genes [hepatocellular carcinoma (HCC) data in Gene Expression Profiling Interactive Analysis (GEPIA) database, *n* = 364]. **(A–I)** Kaplan–Meier curves for overall survival in *ANLN*, *BIRC5*, *BUB1B*, *CDC20*, *CDCA5*, *CDK1*, *NCAPG*, *NEK2*, and *TOP2A* (*P* < 0.01).

**FIGURE 7:**
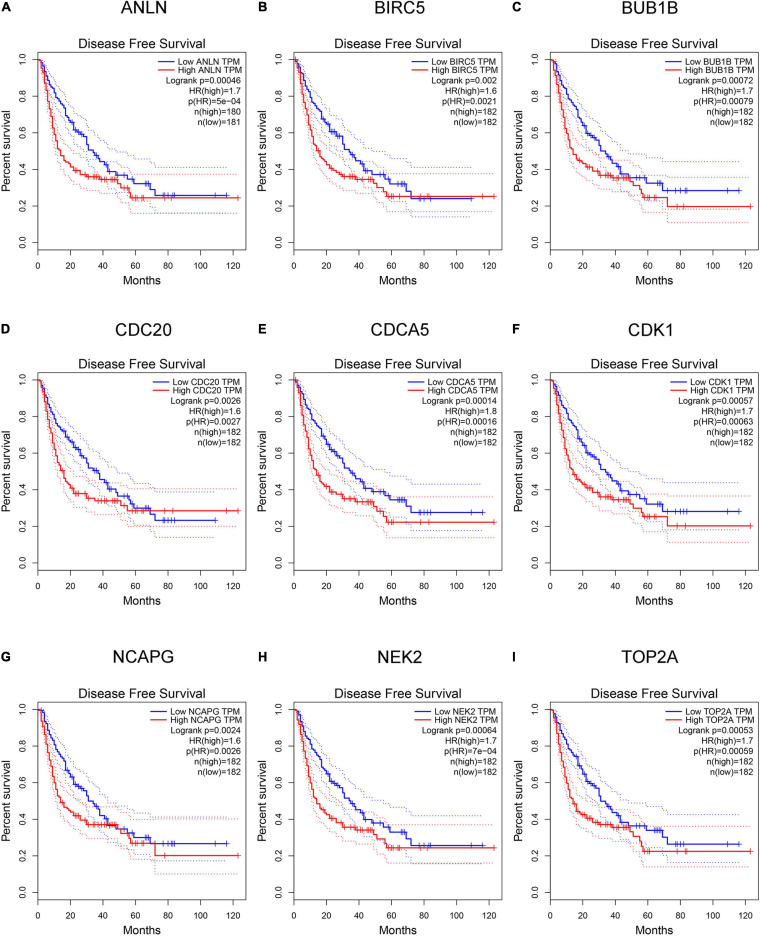
Kaplan–Meier plot of disease-free survival analysis of the nine key genes [hepatocellular carcinoma (HCC) data in Gene Expression Profiling Interactive Analysis (GEPIA) database, *n* = 364]. **(A–I)** Kaplan–Meier curves for disease-free survival in *ANLN*, *BIRC5*, *BUB1B*, *CDC20*, *CDCA5*, *CDK1*, *NCAPG*, *NEK2*, and *TOP2A* (*P* < 0.01).

**FIGURE 8:**
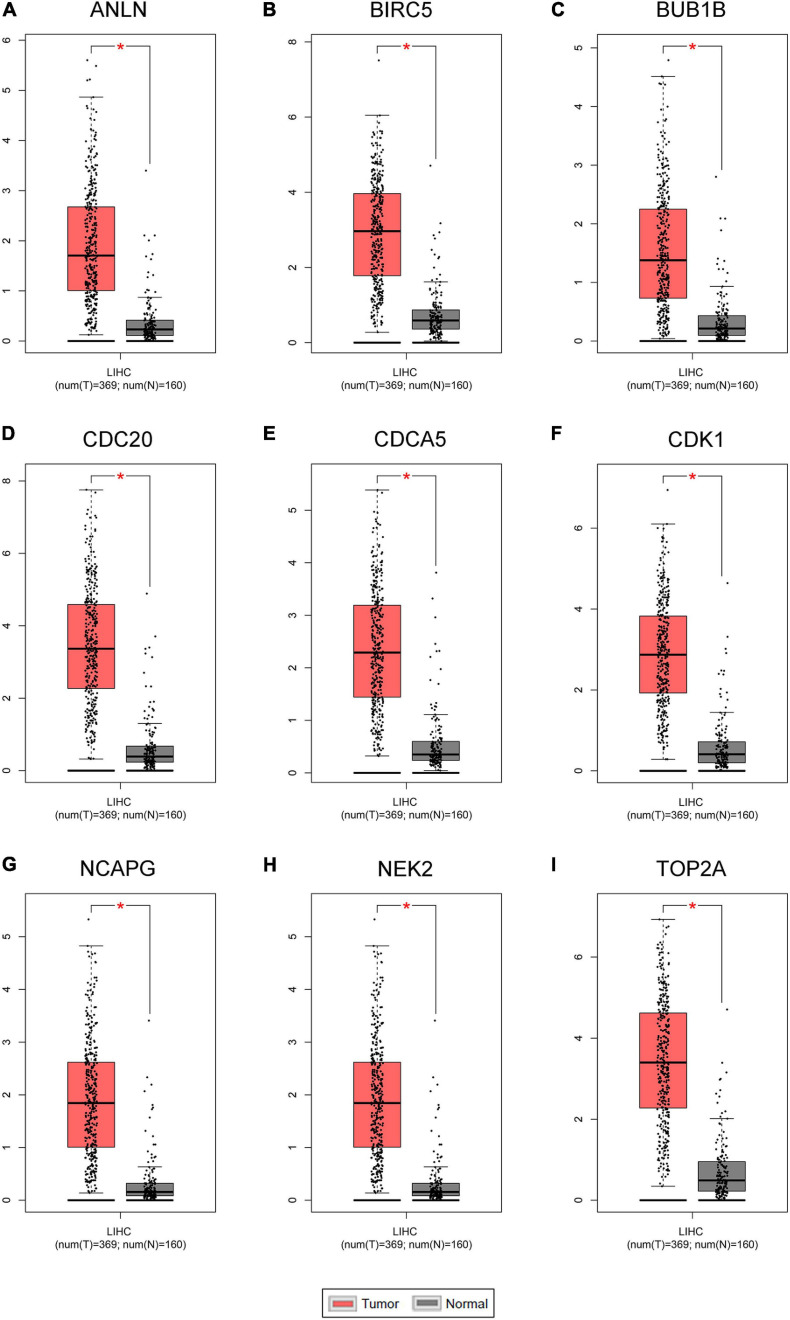
The mRNA expression levels of the nine key genes between normal liver samples and hepatocellular carcinoma (HCC) samples in Gene Expression Profiling Interactive Analysis (GEPIA) HCC database (*n* = 529). **(A–I)** *ANLN*, *BIRC5*, *BUB1B*, *CDC20*, *CDCA5*, *CDK1*, *NCAPG*, *NEK2*, and *TOP2A* are significantly increased in HCC samples compared with normal samples (*P* < 0.01).

**FIGURE 9:**
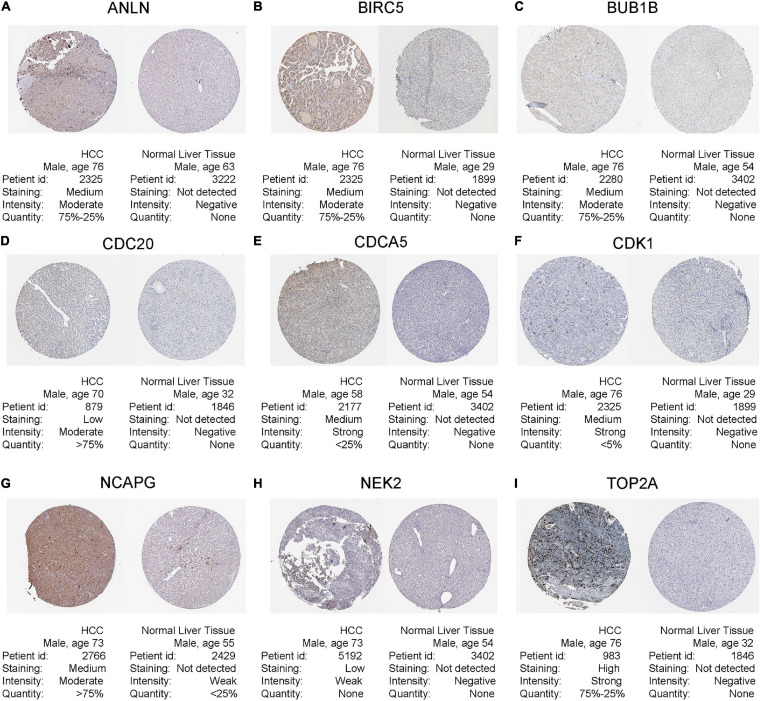
Human Protein Atlas immunohistochemistry of normal sample (N) and tumor sample (T) using **(A)** anti-ANLN, **(B)** anti-BIRC5, **(C)** anti-BUB1B, **(D)** anti-CDC20, **(E)** anti-CDCA5, **(F)** anti-CDK1, **(G)** anti-NCAPG, **(H)** anti-NEK2, and **(I)** anti-TOP2A.

**FIGURE 10:**
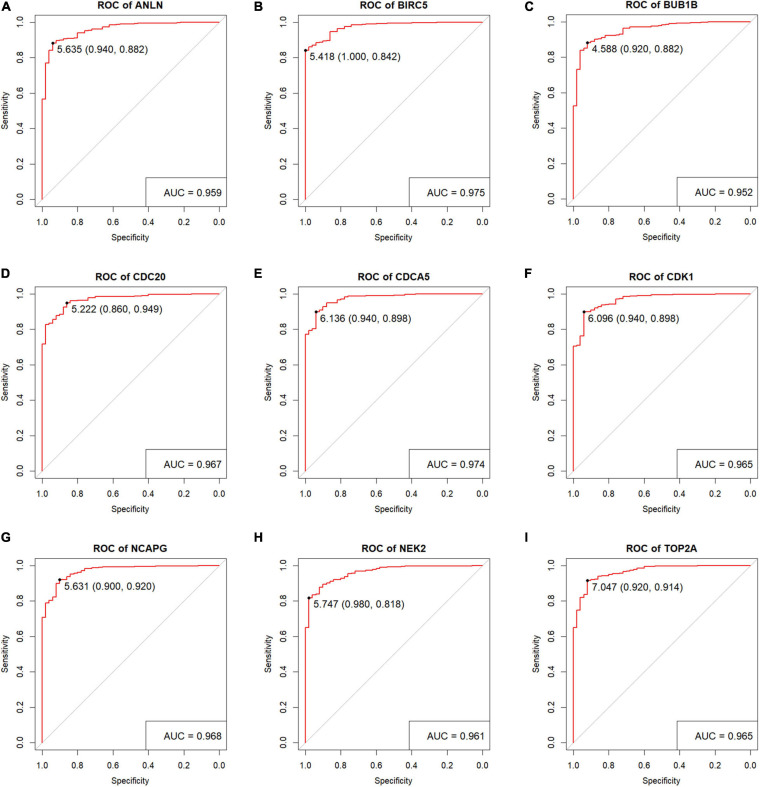
Verification the diagnostic performance of the nine key genes. Receiver operating characteristic (ROC) curves were generated to verify the capacity to differentiate tumor sample from normal sample, showing excellent specificity and sensitivity in The Cancer Genome Atlas (TCGA) hepatocellular carcinoma (HCC) dataset. **(A)** *ANLN*, **(B)** *BIRC5*, **(C)** *BUB1B*, **(D)** *CDC20*, **(E)** *CDCA5*, **(F)** *CDK1*, **(G)** *NCAPG*, **(H)** *NEK2*, and **(I)** *TOP2A*.

**FIGURE 11:**
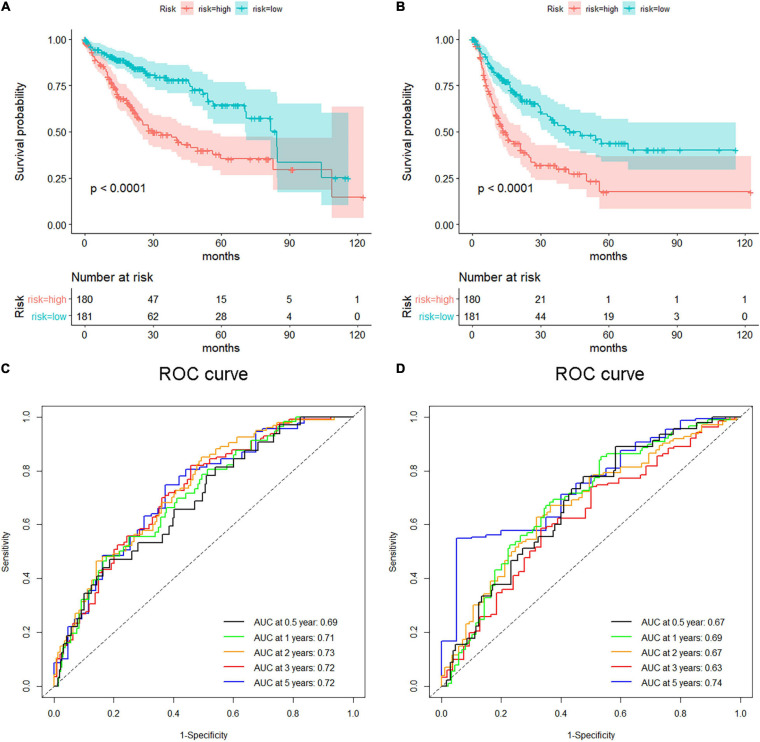
Survival analysis of the nine-gene risk score in validation datasets [The Cancer Genome Atlas (TCGA)]. **(A,B)** Kaplan–Meier overall survival **(A)** and disease-free survival **(B)** curves for patients in TCGA datasets divided into high- and low-risk groups according to the risk score. Patients with a high-risk score showed poorer overall survival and disease-free survival in TCGA hepatocellular carcinoma (HCC) cohorts. Receiver operating characteristic (ROC) curves exhibited the predictive value of the risk signature for HCC patients in TCGA datasets on overall survival **(C)** and disease-free survival **(D)**.

Hepatocellular carcinoma data from the GEPIA database were used to validate 22 hub genes. Among them, *ANLN*, *BIRC5*, *BUB1B*, *CDC20*, *CDCA5*, *CDK1*, *NCAPG*, *NEK2*, and *TOP2A* were most negatively related to overall survival (Figure 6) and disease-free survival (Figure 7) in the Kaplan–Meier survival analysis of HCC patients. Furthermore, the expression profile from GEPIA and the Oncomine database shows that the mRNA expression levels of these nine genes were apparently higher in HCC samples compared with that of normal samples (Supplementary Figure 2 and Figure 8). Moreover, the correlation analysis of gene expression levels and HCC stage based on TCGA HCC data shows that the expressions of these nine genes were gradually upregulated along with the tumor stage increase (Supplementary Figure 3). In addition, immunohistochemistry (IHC) staining of the proteins encoded by these nine key genes, obtained from The Human Protein Atlas database, also showed that the protein levels are significantly upregulated in tumor samples compared with normal samples, which was in accordance with the transcriptional results (Figure 9). ROC curves were plotted to examine the diagnostic capability of these nine genes *via* TCGA HCC data. The area under the curve (AUC) showed that *ANLN*, *BIRC5*, *BUB1B*, *CDC20*, *CDCA5*, *CDK1*, *NCAPG*, *NEK2*, and *TOP2A* showed excellent diagnostic performance on discriminating tumor from normal samples (Figure 10). Finally, a multivariate Cox regression analysis was performed to further evaluate whether it could provide sufficient prognostic capacity according to the expression levels of these nine genes. The results in Table 1 and Figure 11 further confirm that the risk score based on the gene signature for these nine genes had high sensitivity and specificity and was a reliable clinical prognostic factor for HCC patients.

**TABLE 1:** Multivariate Cox regression analysis of potential prognostic factors for HCC patients in validation datasets (TCGA).

Variables	Overall survival			Disease-free survival		
	Hazard ratio	95% CI of hazard ratio	*P*	Hazard ratio	95%CI of hazard ratio	*P*
Age	1.01	0.99–1.02	0.299	1.00	0.99–1.01	0.856
Gender	0.79	0.54–1.14	0.208	1.21	0.84–1.74	0.297
Grade	1.02	0.79–1.32	0.870	1.10	0.87–1.40	0.407
Stage	1.52	1.25–1.85	2.78E-05	1.60	1.32–1.94	1.88E-06
Risk score	1.67	1.37–2.02	1.90E-07	1.79	1.12–2.85	0.014

*HCC, hepatocellular carcinoma; TCGA, The Cancer Genome Atlas.*

### Gene Set Enrichment Analysis

To obtain deeper insight into the function of these nine key genes, GSEA was performed to related KEGG pathways by using TCGA HCC data. Based on the criteria of cutoff (FDR < 0.05, gene size > 10, and ES > 0.65), the GSEA results show that those nine key gene high-expression samples were most enriched in the cell cycle pathway (NES = 2.07, FDR = 0.012, and gene size = 118; Supplementary Figure 4).

### Genetical Alteration Profiles of the Nine Key Genes

**FIGURE 12:**
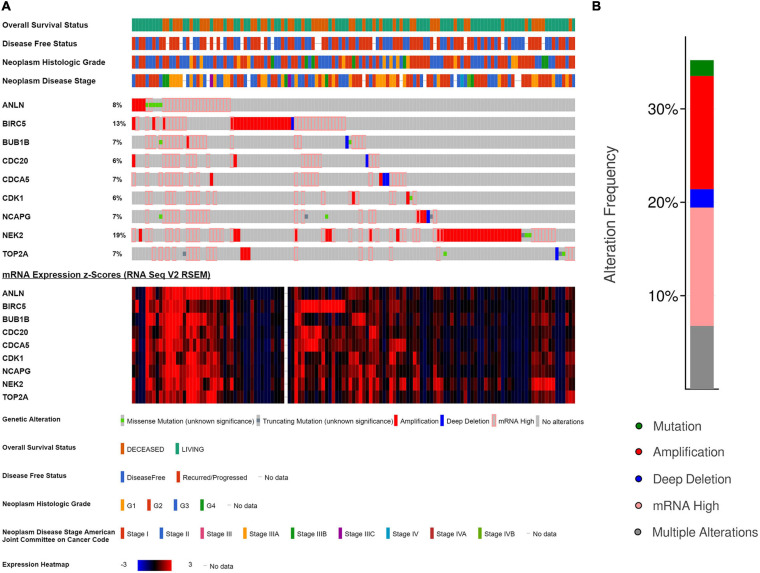
Genetic alterations of the nine key genes in The Cancer Genome Atlas (TCGA)-Hepatocellular Carcinoma (HCC). **(A)** A detailed genetic alteration chart of the nine key genes showing there were 130 (36.11%) genetic alterations in 360 HCC samples. **(B)** The total alteration frequency summary of the nine key genes in TCGA-HCC.

OncoPrint of cBioPortal was used to visualize the nine key genes’ alteration condition in TCGA HCC patients, showing that the nine key genes were altered in 130 (36.11%) of 360 HCC patients (Figure 12B), and the detailed alteration status of each gene was shown in Figure 12A. *NEK2* and *BIRC5* were the most altered genes (19 and 13%, respectively), with mRNA upregulation and amplification being the major types (12.22 and 13.33%, respectively).

### Related Small-Molecule Drug Screening for High-Risk Hepatocellular Carcinoma

To identify candidate small molecules for high-risk HCC, CMap, a systematic bioinformatics algorithm, was applied to identify functional connections between small-molecule drugs and gene expression signatures. Using DEGs (| log2FC| ≥ 1, FDR < 0.05) in the turquoise module as query, 10 small-molecule drugs related to high-risk HCC were identified (instances > 10, *P* value < 0.05, and Enrichment < 0; Table 2). Among these small molecules, vorinostat, alpha-estradiol, trichostatin-A, trifluoperazine, and tretinoin exhibited relatively higher negative correlation and, therefore, showed a potential therapeutic value against HCC.

**TABLE 2:** Results of CMap analysis.

Rank	CMap name	Mean	Instances (n)	Enrichment	P value	Specificity score
1	Vorinostat	−0.564	12	−0.449	0.00964	0.4956
2	Alpha-estradiol	−0.49	16	−0.389	0.01109	0.2077
3	Trichostatin-A	−0.479	182	−0.298	0	0.5941
4	Trifluoperazine	−0.479	16	−0.419	0.00479	0.1827
5	Tretinoin	−0.452	22	−0.293	0.0362	0.25
6	Thioridazine	−0.435	20	−0.315	0.02852	0.4194
7	Tanespimycin	−0.417	62	−0.224	0.00338	0.5462
8	Chlorpromazine	−0.409	19	−0.33	0.02381	0.1026
9	LY-294002	−0.345	61	−0.205	0.00986	0.6258
10	Sirolimus	−0.332	44	−0.222	0.02208	0.5685

## Discussion

Hepatocellular carcinoma is one of the most life-threatening malignant tumors in the world. Biomarkers with higher predictive accuracy, particularly for small-molecule target drugs, are urgently needed for better prognosis and clinical treatment in patients with HCC. In this study, we selected three publicly available HCC cohorts in GEO and identified 1,704 common DEGs between HCC samples and normal samples. WGCNA was then performed using these DEGs, the co-expression gene module most closely related to the stage of HCC was identified, and 22 hub genes were screened. Subsequently, after screening using the Kaplan–Meier data in the GEPIA database, we identified nine key genes related to progression and prognosis of HCC patients. Moreover, to validate these nine genes, we performed multivariate Cox analysis using data from TCGA HCC database and confirmed the gene expression dysregulation by the GEPIA database, Oncomine database, and cBioPortal database and protein expression from the Human Protein Atlas. In addition, using the CMap database, several drugs, with the potential to treat HCC, were identified.

The nine key genes are composed of *ANLN*, *BIRC5*, *BUB1B*, *CDC20*, *CDCA5*, *CDK1*, *NCAPG*, *NEK2*, and *TOP2A*. They have been shown to be oncogenes, mainly enriched in cell division and cell cycle pathways, and capable of influencing HCC progression and prognosis. Anillin actin binding protein (ANLN), an actin-binding protein, is instrumental in cell growth and migration and in cell division. Zhang et al. (2018) reported that the dysregulation of *ANLN* can block cell division in human liver cells and prevent the development of liver tumors in mice. Also, Magnusson et al. (2016) reported that the expression level of *ANLN* in tumor cells is highly associated with poor prognosis in breast cancer patients and can be regarded as a potential independent prognostic biomarker. Baculoviral IAP repeat containing 5 (BIRC5), also called survivin, as a member of the inhibitors of apoptosis proteins (IAP) family, can inhibit apoptosis and promote cell proliferation. It was reported that *BIRC5* was highly upregulated in HCC cells, exerting strong antiapoptotic effect, promoting cell proliferation, and enhancing the HCC cell resistance to radiation (Jin et al., 2014; Su, 2016). Mitotic checkpoint serine/threonine kinase B (*BUB1B*) encodes a kinase involved in spindle checkpoint functions. Fu et al. (2016) reported that BUB1B exerts a crucial effect in tumor development and progression *via* regulating the proliferation, migration, and invasion in prostate cancer cells. And Zhuang et al. (2018) suggested that upregulation of *BUB1B* in HCC samples indicates poor overall survival and disease-free survival in HCC patients and could be a novel therapeutic target for HCC treatment. Multiple cell cycle genes were identified, including *CDC20*, *CDCA5*, and *CDK1*. CDC20 is a regulatory protein in the cell cycle checkpoint. A meta-analysis based on 1,856 patients suggested that high-level CDC20 expression indicated poor prognosis (Wang et al., 2018). Wu et al. (2013) reported that the expression level of *CDC20* was an independent prognostic factor and could be a potential prognostic biomarker of human colorectal cancer. Chang et al. (2012) also found that abnormal *CDC20* expression may exert an important effect in pancreatic ductal adenocarcinoma tumorigenesis and progression and *CDC20* may serve as a biomarker of cancer progression and prognosis. Cell division cycle associated 5 (*CDCA5*) encoding sororin is an essential cell cycle-dependent regulator of sister chromatid cohesion (Rankin, 2005). Shen et al. (2018) reported that knockdown of *CDCA5* significantly inhibited HCC cell proliferation and suppressed cell survival and, thereby, could be a potential therapeutic target for HCC. Additionally, a 178-patient retrospective study found that overexpression of *CDCA5* was highly related to poor prognosis in patients with HCC (Tian et al., 2018). Cyclin-dependent kinase 1 (CDK1), a Ser/Thr protein kinase, is a catalytic subunit of M-phase promoting factor (MPF), which plays a key role in G1 progress, G1–S transition, and G2–M transitions in the eukaryotic cell cycle process (Malumbres and Barbacid, 2009). Many studies reported that *CDK1* expression level was directly proportional to tumor grade and poor patient outcomes in pancreatic ductal adenocarcinoma, lung adenocarcinoma, and HCC (Shi et al., 2016; Piao et al., 2019; Zhou et al., 2019). Non-SMC Condensin I Complex Subunit G (*NCAPG)*, encoding a subunit of the condensin complex, plays a key role in chromosome condensation and stabilization in the process of cell division. A genome-wide CRISPR (clustered regularly interspaced short palindromic repeats) knockout study identified that *NCAPG* was an important oncogene for HCC tumor development (Wang et al., 2019). NIMA Related Kinase 2 (*NEK2*), encoding a serine/threonine protein kinase, was involved in centrosome separation and bipolar spindle formation during mitosis. Previous studies reported that overexpression of *NEK2* contributes to invasion and metastasis of HCC, which was related to poor prognosis, suggesting that *NEK2* could be a potential prognostic biomarker for HCC (Li et al., 2017). Lu et al. (2015) reported that overexpression of NEK2 indicates the malignant behavior of colon cancer and has diagnostic and prognostic value in colon cancer. DNA Topoisomerase II Alpha (*TOP2A*), encoding a DNA topoisomerase, controls topologic states of DNA during transcription. Previous studies reported that overexpression of *TOP2A* promoted the progression of breast cancer and prostate cancer (Kirk et al., 2015; Shigematsu et al., 2018).

The survival analysis and the multivariate Cox regression results support that these nine key genes identified in our study could be potential biomarkers for predicting prognosis for HCC patients. Moreover, to further understand the potential mechanisms involved with these key genes, GSEA was performed using validation datasets (TCGA HCC), showing that the cell cycle pathway was the most significant term. It has been reported that cell cycle and cell death processes were regulated by complex pathways; disruption of these vital pathways may lead to uncontrolled cell growth, especially in the development process of cancer (Wiman and Zhivotovsky, 2017). And Bai et al. (2017) reported that dysregulation of the cell cycle is a hallmark of tumorigenesis and tumor progression. Therefore, we suppose that dysregulation of the nine genes may play a vital role in HCC development and progression *via* regulating the cell cycle pathway, eventually leading to poor prognosis of HCC.

Connectivity Map 2.0 was applied to predict several small-molecule compounds with potential therapeutic value against HCC using the gene signatures developed here. According to literature, some compounds have already been reported to have anticancer effects, such as vorinostat, trichostatin A, tanespimycin, trifluoperazine, and chlorpromazine. Vorinostat was the first histone deacetylase inhibitor (HDI) authorized by the Food and Drug Administration of the United States for cutaneous T-cell lymphoma (CTCL) treatment in 2006, showing great effect on inducing cancer cell death, reducing angiogenesis, and modulating the immune response (Duvic and Dimopoulos, 2016; Eckschlager et al., 2017). Trichostatin A is also an inhibitor of histone deacetylases, showing antitumor capacity by activating classic and alternative cell death signaling pathways. Diamantis (2018) reported that trichostatin A exerted a potential therapeutic effect by epigenetic regulation in the treatment of HCC (Tsilimigras et al., 2018). Tanespimycin is an antibiotic that has been studied to use for treating many diseases, such as lung injury, sepsis, and cancer, specifically for breast cancer (Modi et al., 2011), leukemia (Dimopoulos et al., 2011), ovarian carcinoma (Hendrickson et al., 2012), and multiple myeloma (Richardson et al., 2010). Trifluoperazine is a typical antipsychotic. It is reported that trifluoperazine could effectively restrict angiogenesis and tumor growth in HCC (Jiang et al., 2017). It is reported that chlorpromazine also shows an anticancer function by inhibiting the growth and proliferation of chemoresistant glioma cells (Oliva et al., 2017). Therefore, with the above literature exploration and the results based on the bioinformatics and CMap analysis, we suggest that these identified small-molecule drugs could have potential therapeutic value to treat HCC.

In this study, there were some limitations. First, all of the data for analysis, explorations, and validations were obtained from public databases. Consequently, a controlled multicenter experimental study will be needed to validate the results of the study. Second, the nine key genes need to be studied at the cellular level to explore molecular mechanisms between these genes and HCC malignant characteristics.

In summary, by using WGCNA and bioinformatics analyses, nine key genes were identified as involved in HCC progression and prognosis. The cell cycle pathway was the core pathway enriched with these key genes. Several candidate molecule drugs with the potential to reverse the effects of these genes in HCC tumors were also identified, providing potential HCC targeted therapy.

## Data Availability Statement

The original contributions presented in the study are included in the article/Supplementary Material, further inquiries can be directed to the corresponding author/s.

## Author Contributions

NJ, XZ, and JW: conceptualization. NJ, FK, DQ, and AW: methodology. NJ, DQ, and JY: software. LW and YS: validation. HL and JL: formal analysis. NJ, XZ, and XS: investigation. HL and AW: data curation. NJ and XZ: writing–original draft preparation. FK and JW: writing–review and editing and supervision. JY and XS: visualization. JW: funding acquisition. All authors have read and agreed to the published version of the manuscript.

## Conflict of Interest

The authors declare that the research was conducted in the absence of any commercial or financial relationships that could be construed as a potential conflict of interest.

## Footnotes

1 http://www.ncbi.nlm.nih.gov/geo/

2 https://xenabrowser.net/datapages/

3 https://david.ncifcrf.gov/summary.jsp

4 http://www.cbioportal.org/

5 https://www.gsea-msigdb.org/gsea/msigdb

6 https://portals.broadinstitute.org/cmap

## Supplementary Material

Supplementary Figure 1 Clustering dendrogram of 35 HCC samples with clinical traits and soft-thresholding power determination. **(A)** The clustering was based on the expression data of the common DEGs in HCC (*n* = 35). The red color intensity was directly proportional to HCC stage. **(B)** Analysis of network topology of the scale-free fit index for various soft-thresholding powers. **(C)** Analysis of network topology of the mean connectivity for soft-thresholding powers. **(D,E)** Checking the scale free topology when soft-thresholding power β = 4.

Supplementary Figure 2 Rank of gene expression of nine key genes in Oncomine database.

Supplementary Figure 3 Verification of the correlation between mRNA expression levels and the stages of HCC based on HCC data in TCGA database (*n* = 360). **(A)** *ANLN*, **(B)** *BIRC5*, **(C)** *BUB1B*, **(D)** *CDC20*, **(E)** *CDCA5*, **(F)** *CDK*, **(G)** *NCAPG*, **(H)** *NEK2*, and **(I)** *TOP2A*.

Supplementary Figure 4 Gene set enrichment analysis on the basis of TCGA HCC cohort. The GSEA result shows those nine key genes high expression HCC samples were most enriched in the cell cycle pathway (NES = 2.07, FDR = 0.012, and gene size = 118).

Supplementary Table 1 Public gene expression profile datasets used in this study.

Supplementary Table 2 Turquoise module genes.
