# Supplementary figures and images for: Identification of Core Genes Related to Progression and Prognosis of Hepatocellular Carcinoma and Small-Molecule Drug Predication
Source: Front Genet. 2021 Feb 23;12:608017. doi: 10.3389/fgene.2021.608017 (PMC7940693; doi:10.3389/fgene.2021.608017)

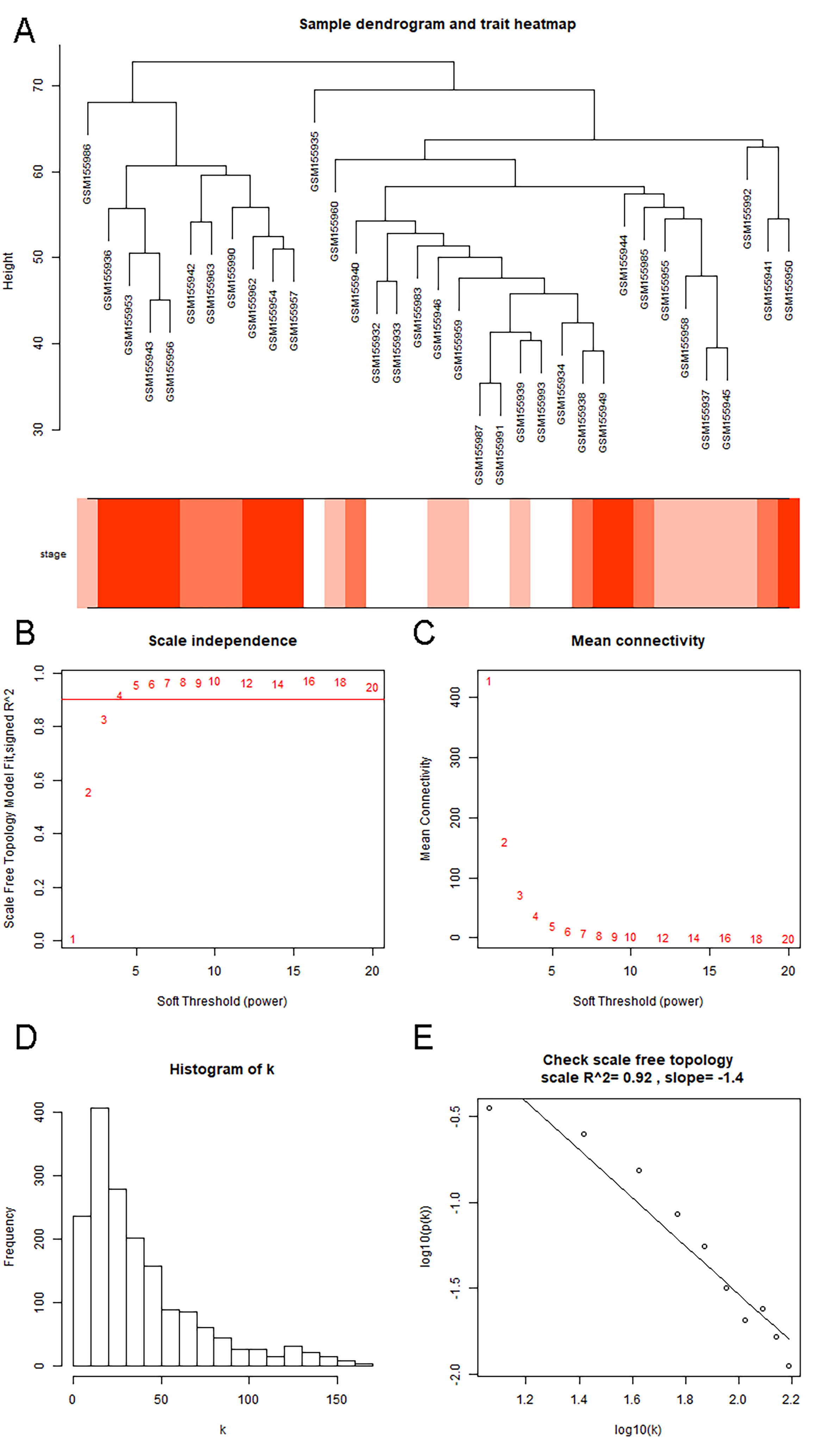

Supplement: Supplementary Figure 1 — Clustering dendrogram of 35 HCC samples with clinical traits and soft-thresholding power determination. (A) The clustering was based on the expression data of the common DEGs in HCC (n = 35). The red color intensity was directly proportional to HCC stage. (B) Analysis of network topology of the scale-free fit index for various soft-thresholding powers. (C) Analysis of network topology of the mean connectivity for soft-thresholding powers. (D,E) Checking the scale free topology when soft-thresholding power β = 4. [file Image_1.JPEG]

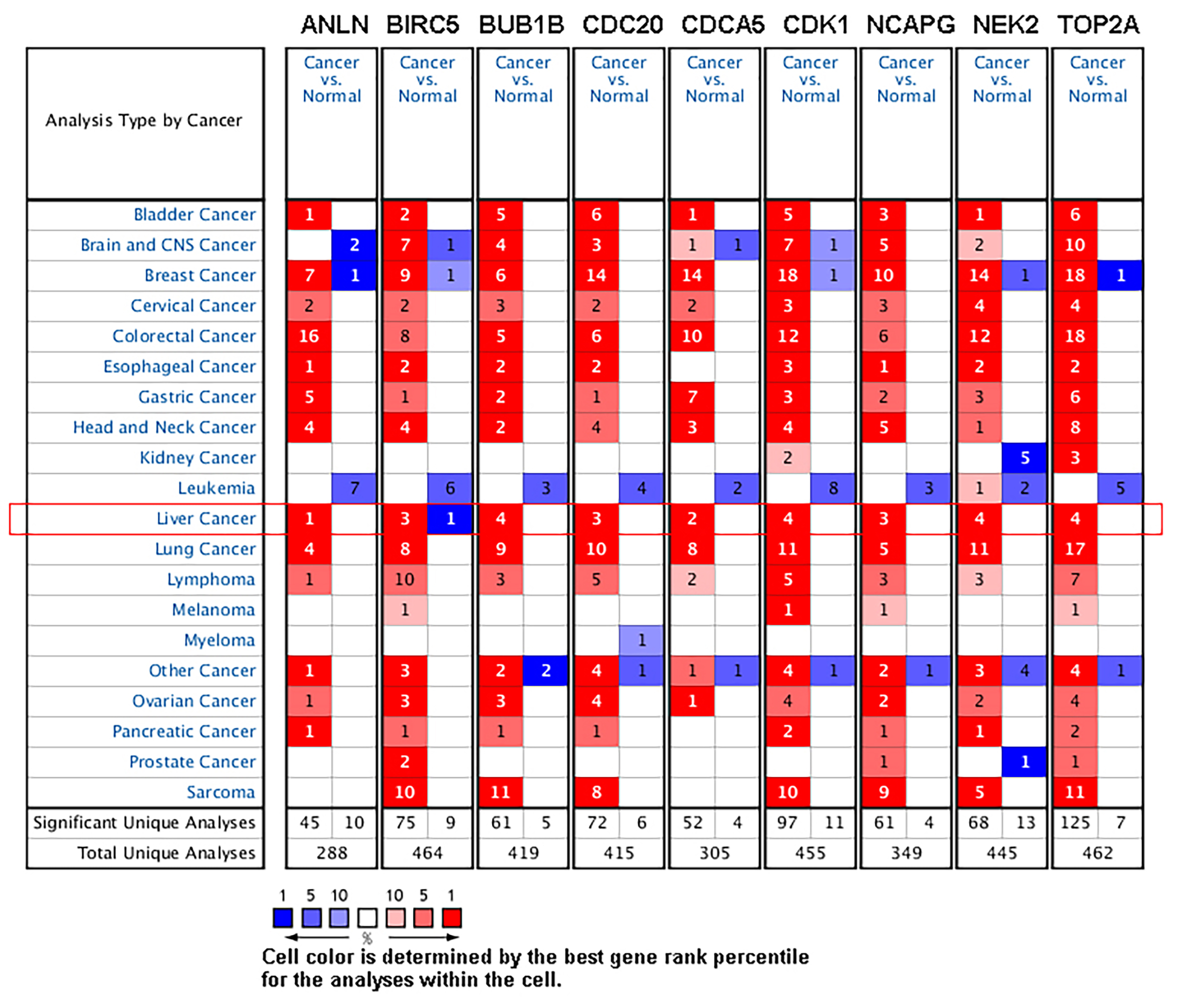

Supplement: Supplementary Figure 2 — Rank of gene expression of nine key genes in Oncomine database. [file Image_2.JPEG]

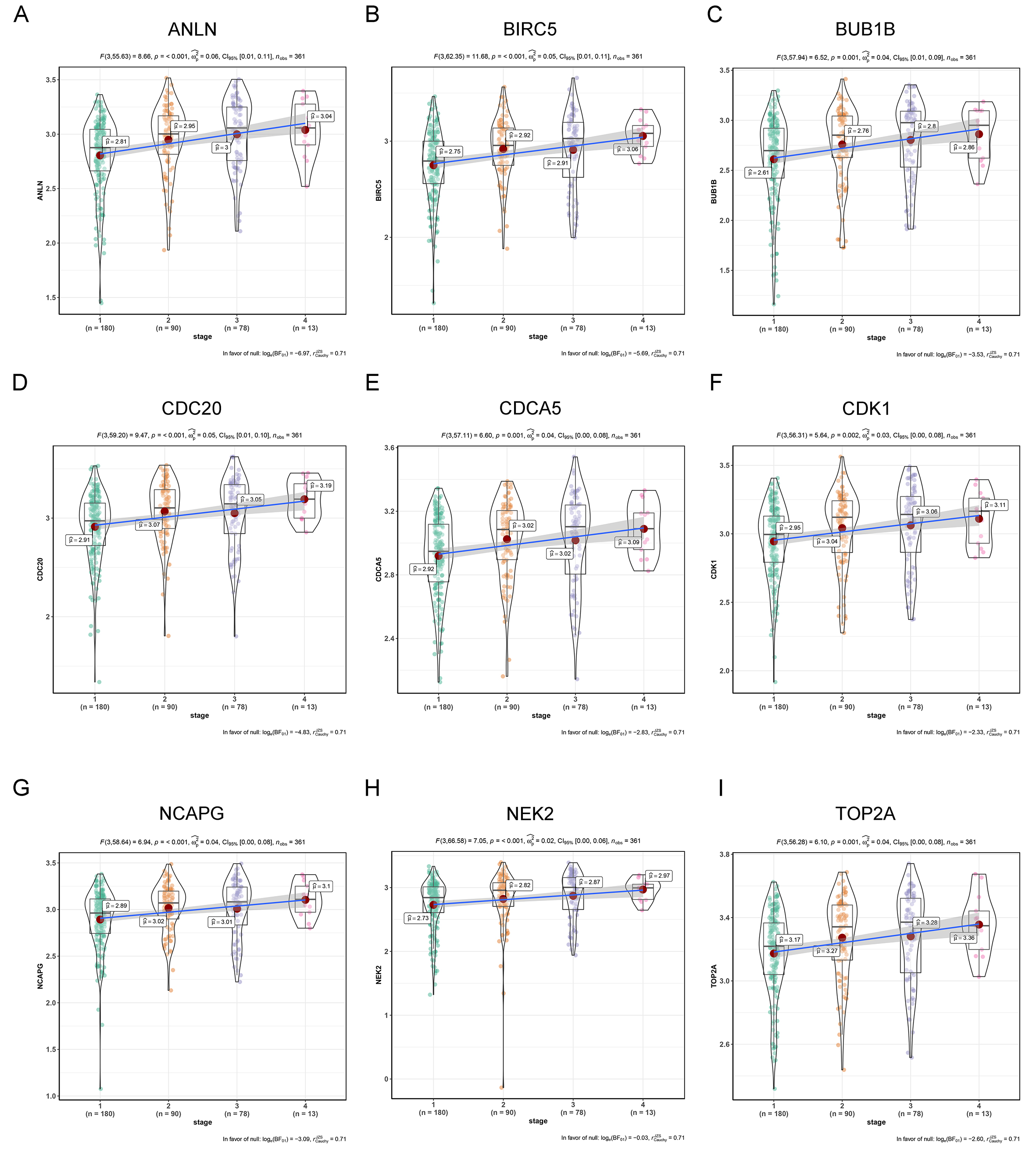

Supplement: Supplementary Figure 3 — Verification of the correlation between mRNA expression levels and the stages of HCC based on HCC data in TCGA database (n = 360). (A) ANLN, (B) BIRC5, (C) BUB1B, (D) CDC20, (E) CDCA5, (F) CDK, (G) NCAPG, (H) NEK2, and (I) TOP2A. [file Image_3.JPEG]

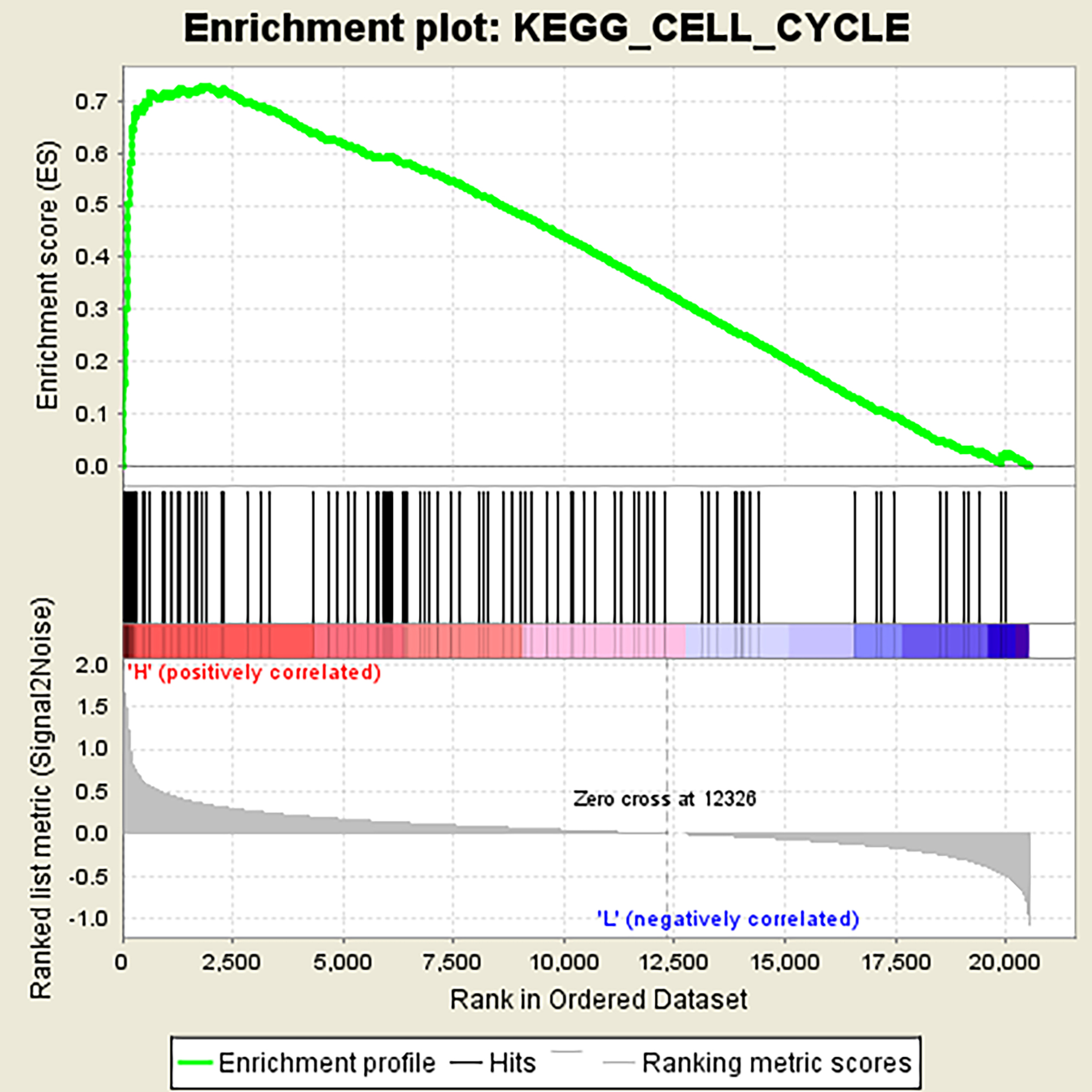

Supplement: Supplementary Figure 4 — Gene set enrichment analysis on the basis of TCGA HCC cohort. The GSEA result shows those nine key genes high expression HCC samples were most enriched in the cell cycle pathway (NES = 2.07, FDR = 0.012, and gene size = 118). [file Image_4.JPEG]
